# Viral expression of ALS-linked ubiquilin-2 mutants causes inclusion pathology and behavioral deficits in mice

**DOI:** 10.1186/s13024-015-0026-7

**Published:** 2015-07-08

**Authors:** Carolina Ceballos-Diaz, Awilda M. Rosario, Hyo-Jin Park, Paramita Chakrabarty, Amanda Sacino, Pedro E. Cruz, Zoe Siemienski, Nicolas Lara, Corey Moran, Natalia Ravelo, Todd E. Golde, Nikolaus R. McFarland

**Affiliations:** Center for Translational Research in Neurodegenerative Disease, Department of Neuroscience, University of Florida, 1275 Center Dr, PO Box 100159, Gainesville, FL 32610 USA; Department of Neurology, College of Medicine, University of Florida, 1149 S Newell Dr, L3-100, PO Box 100236, Gainesville, FL 32610 USA

**Keywords:** Ubiquilin-2, Amyotrophic lateral sclerosis (ALS), Proteinopathy, Somatic brain transgenesis, Mouse model

## Abstract

**Background:**

*UBQLN2* mutations have recently been associated with familial forms of amyotrophic lateral sclerosis (ALS) and ALS-dementia. *UBQLN2* encodes for ubiquilin-2, a member of the ubiquitin-like protein family which facilitates delivery of ubiquitinated proteins to the proteasome for degradation. To study the potential role of ubiquilin-2 in ALS, we used recombinant adeno-associated viral (rAAV) vectors to express UBQLN2 and three of the identified ALS-linked mutants (P497H, P497S, and P506T) in primary neuroglial cultures and in developing neonatal mouse brains.

**Results:**

In primary cultures rAAV2/8-mediated expression of UBQLN2 mutants resulted in inclusion bodies and insoluble aggregates. Intracerebroventricular injection of FVB mice at post-natal day 0 with rAAV2/8 expressing wild type or mutant UBQLN2 resulted in widespread, sustained expression of ubiquilin-2 in brain. In contrast to wild type, mutant UBQLN2 expression induced significant pathology with large neuronal, cytoplasmic inclusions and ubiquilin-2-positive aggregates in surrounding neuropil. Ubiquilin-2 inclusions co-localized with ubiquitin, p62/SQSTM, optineurin, and occasionally TDP-43, but were negative for α-synuclein, neurofilament, tau, and FUS. Mutant UBLQN2 expression also resulted in Thioflavin-S-positive inclusions/aggregates. Mice expressing mutant forms of UBQLN2 variably developed a motor phenotype at 3–4 months, including nonspecific clasping and rotarod deficits.

**Conclusions:**

These findings demonstrate that UBQLN2 mutants (P497H, P497S, and P506T) induce proteinopathy and cause behavioral deficits, supporting a “toxic” gain-of-function, which may contribute to ALS pathology. These data establish also that our rAAV model can be used to rapidly assess the pathological consequences of various *UBQLN2* mutations and provides an agile system to further interrogate the molecular mechanisms of ubiquilins in neurodegeneration.

## Background

Several mutations in the *UBQLN2* gene have recently been identified and associated with X-linked familial ALS and ALS-dementia [1–3]. *UBQLN2* encodes ubiquilin-2, a member of the ubiquitin-like family of proteins that facilitate delivery of polyubiquitinated proteins to the proteasome for degradation [1]. In humans there are at least 4 ubiquilins. Each is widely expressed, except for ubiquilin-3 which is testes specific [4]. Ubiquilins are characterized by an N-terminal ubiquitin-biding domain (UBA), a variable number of Sti1-like repeats, and a C-terminal ubiquitin-like domain (UBL) that associates with the proteasome. Identified ALS-linked mutations (P497S/H, P506TS/T, and P525S) are primarily located in a C-terminal proline-rich domain that contains 12 PXX repeats [1]; however, 3 have been identified outside this region [2]. Recently, another mutation was identified within the proline-rich region in *UBQLN2* and linked to familial ALS (c.1490C > T, p.P497L) [3]. Mutations in ubiquilin-2 have been proposed to alter proteasome mediated protein clearance, suggesting a loss-of-function and possible cause for abnormal protein accumulation and deposition [1]. However, ubiquilins have also been implicated in ER-associated protein degradation and autophagy [5–7]. Examination of protein inclusions in pathological tissue from both sporadic ALS and ALS-dementia demonstrate the presence of ubiquilin-2 in inclusions and co-localization with other proteins such as ubiquitin and p62/SQSTM1, further suggesting a role for ubiquilin-2 in proteinopathy and in ALS pathology [1, 8, 9]. Few studies to date, however, have examined the role of ubiquilin-2 and consequence of identified mutations—so far limited to P497H mutant—on the development of ALS pathology [10, 11].

To determine the pathological consequences of *UBQLN2* mutants, we developed rAAV 2/8 vectors to compare the effects of overexpression of wild type (WT) and three of the recently identified ALS-mutant ubiquilins in primary neuroglial cultures and in the developing mouse brain. In mice we utilized “somatic brain transgenesis” (SBT) to rapidly introduce and express *UBQLN2* mutants in throughout the brain. Although having more limited and variable expression compared to traditional transgenic models, SBT still allows for rapid, widespread expression and screening of genes of interest before expending the time and expense developing traditional transgenic models [12, 13]. Our findings demonstrate that overexpression of pathological forms of mutant ubiquilin-2 compared to WT all develop widespread inclusion pathology, including amyloid-like aggregates, that persists over 6 months and which is associated with mild, early motor deficits. These studies provide further insight into the *in vivo* effects of expression of ALS-linked mutant forms of ubiquilin-2 in mice. Furthermore, our SBT mouse models demonstrate a powerful and complementary approach to traditional transgenics that will allow further dissection of pathological mechanisms of ubiquilin-2 mutants and their role in development of ALS and ALS-dementia.

## Results

To study the effects of recently described ALS-linked *UBQLN2* mutants on pathology we cloned wild-type (WT) and three mutant forms of ubiquilin-2 (P497S, P497H and P506T) into rAAV vectors for expression in developing mouse brain. Viral expression was first tested in primary neuroglial cultures before moving to mice.

### Viral expression of ubiquilin-2 mutants in mixed neuroglia cultures results in large punctate intracellular accumulations

**Fig. 1:**
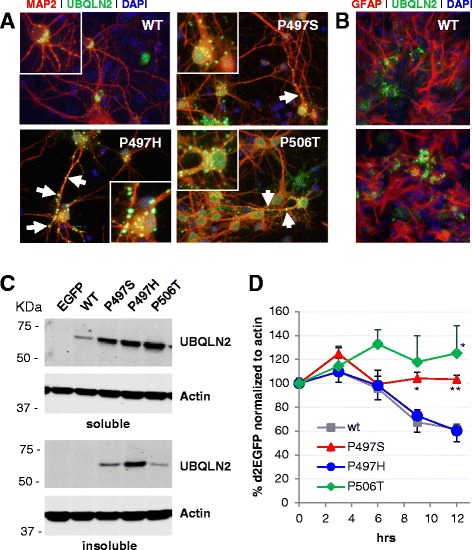
Mutant Ubiquilin-2 overexpression results in punctate intracellular accumulations in primary mixed neuroglia cultures. **a**. Cells transduced with AAV-*UBQLN2*(WT) show ubiquilin-2 immunoreactivity (*green*) diffusely present in the cytoplasm and cell processes with few small punctate accumulations. In contrast, *UBQLN2* mutants (P497S, P497H, and P506T) result in large intracellular ubiquilin-2 accumulations both in neuronal soma and processes (red, labelled with MAP2). Ubiquilin-2 accumulations in processes have a “bead on a string”-like appearance particularly for P497H and P506T mutants. **b**. Some ubiquilin-2 accumulations (*green*) are outside of neurons and colocalized with astrocytes in culture, labeled with GFAP-imunoreactivity (*red*). **c**. Western blot of TX-soluble and insoluble fractions show that all *UBQLN2* mutants and not WT accumulate in the TX-insoluble/SDS fraction, suggesting formation insoluble aggregates. **d**. Graph of d2EGFP signal normalized to actin in HEK293 cells transfected with WT and mutant ubiquilin-2. Both P497S and P506T mutants show impaired proteasomal degradation of the d2EGFP reporter compared to the P497H mutant and WT ubiquilin-2. *p < 0.05, **p < 0.01

Recombinant AAV2/8 expressing ubiquilin-2 WT or ALS-linked mutants (P497S, P497H and P506T) was used to transduce primary neuroglial cultures at DIV + 6. Four days post-transduction cells were analyzed by immunofluorescence and biochemistry. Neurons were identified by MAP2 and astrocytes by GFAP co-immunostaining. Ubiquilin-2 expression was primarily observed in neurons in E16 cultures, but also seen in some astrocytes. In cells expressing ubiquilin-2 WT or pathologic mutants, there was low level of diffuse ubiquilin-2 immunoreactivity throughout the neuronal perikarya. Most notably, large accumulations of ubiquilin-2 were seen in both the neuronal cytoplasm and processes. Intracellular ubiquilin-2-postive accumulations, although present in cultures transduced with AAV-*UBQLN2*(WT), were larger and more prevalent in cultures transduced with mutant *UBQLN2* (Fig. 1a). Also, neurons transduced with either P497S, P497H or P506T mutant ubiquilin-2 displayed frequent ubiquilin-2-postive punctate accumulations in neuronal processes with a “bead on a string”-like appearance, suggesting an altered subcellular distribution. These puncta were more apparent in cultures transduced with the P497H and P506T *UBQLN2* mutants and associated with apparent dystrophic changes in neurites. Some ubiquilin-2-postive accumulations were located outside neurons and colocalized with the astrocytic marker GFAP, but not the microglial marker Iba-1 (Fig. 1b). Preliminary screen to identify subcellular localization of intracellular ubiquilin-2 accumulations revealed no colocalization with early endosomal markers such as EEA1 or Rab5; late endosomes, Rab7; autophagosomes, LC3; or lysosomes, LAMP1 (data not shown).

To further assess viral expression of WT and mutant ubiquilin-2 in primary cultures, we performed Western blots on fractionated cell lysates. Notably, mutant forms of ubiquilin-2, but not WT, accumulated in the SDS soluble fraction suggesting that ALS-linked mutant ubiquilins form Triton X-100 (TX) insoluble aggregates (Fig. 1c). As mutations in ubiquilin-2 have been suggested to reduce proteasomal degradation [1], we investigated the effect of expression of different ALS-linked mutant ubiquilin-2 on UPS function in HEK293 cells using the reporter d2EGFP. Twenty-four hours post transfection cells were treated with cyclohexamide and then harvested at 3 h intervals and assessed for d2EGFP levels which were normalized to β-actin. Expression of both the P497S and P506T mutants significantly reduced the rate of d2EGFP proteasomal degradation relative to WT ubiquilin-2 (Fig. 1d). Interestingly, the P497H mutant showed no change in d2EGFP degradation compared to WT in contrast to that previously reported [1].

### SBT expression of ubiquilin-2 mutants results in widespread inclusion pathology

**Fig. 2:**
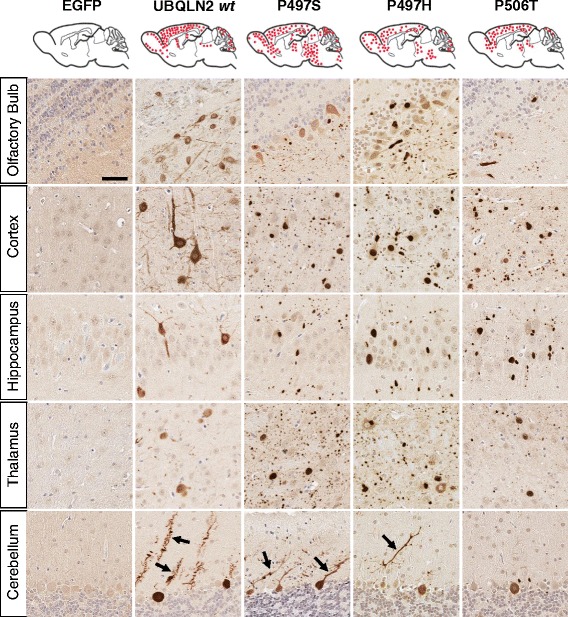
Viral expression of ubiquilin-2 at 6 months. Representative schema of sagittal section shows the overall distribution of AAV-UBQLN2 expression in mouse brain after ICV injection (SBT model). Photos show ubiquilin-2 immunostaining in representative sections animals injected with AAV expressing either EGFP control or WT vs P497S, P497H, or P506T mutant ubiquilin-2. WT ubiqulin-2 expression is homogeneous throughout the neuronal perykaria, including processes. Mutant ubiquilin-2 shows altered subcellular expression, often concentrating in the nucleus, but also resulting cytoplasmic inclusions. Ubiquilin-2-positive “aggregates” are seen also in adjacent neuropil. Arrows point out alteration in purkinje cell dendritic arbors for mutant vs WT ubiquilin-2. (Scale same for all photomicrographs, bar = 50 μm)

**Fig. 3:**
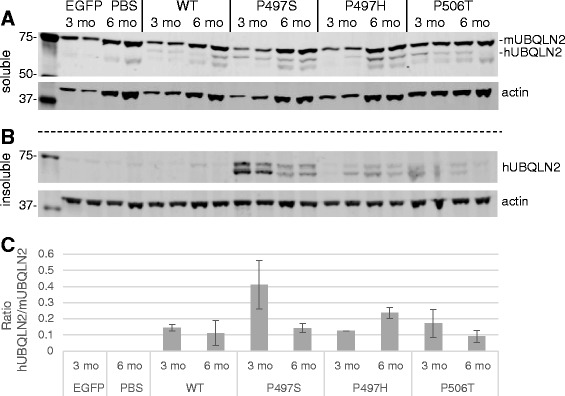
Viral expression of human ubiquilin-2 in whole brain lysates. Western blots of brain lysates from 3 and 6 month animals demonstrate sustained expression of WT ubiquilin-2 and mutants. In the Trition-X100 soluble fraction (**a**) three bands are seen for ubiquilin-2: top is mouse UBQLN2 whereas middle and lower (truncated?) bands represent human UBQLN2. **b**) Only mutant forms of human UBQLN2 are seen in the Triton insoluble fractions. **c**) Graph of human vs endogenous mouse UBQLN2 expression in whole brain (Triton soluble) lysates. *N* = 2–3 sample each with mean ± SD ratio shown

**Fig. 4:**
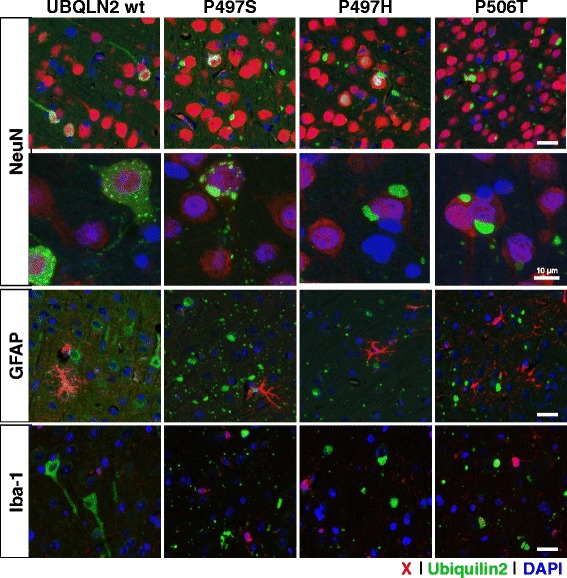
AAV expression of *UBQLN2* WT and mutants is specific to neurons. Images are merged photos of representative cortical areas from 6 month mice stained with immunofluorescence for NeuN/GFAP/Iba-1 (*red*), ubiquilin-2 (*green*), and DAPI (*blue*). The top 2 rows show colocalization of ubiquilin-2 accumulations with NeuN-positive neurons. Row 2 includes high-power confocal images that demonstrate differences in the distribution of ubiquilin-2-containing inclusions in NeuN labeled neurons; large inclusions are seen for all mutant forms in contrast to WT ubiquilin-2. Glial markers GFAP (row 3) and Iba-1 (row 4) rarely colocalize with ubiquilin-2. (Scale bar = 50 μm unless otherwise noted)

To investigate the role of *UBLQN2* and ALS-liked mutations in pathology, we used somatic brain transgenesis with rAAV serotype 2/8 to express either EGFP-control, WT or one of three different mutant forms of ubiquilin-2 (P497S, P497H, and P506T) in the developing mouse brain. Non-transgenic FVB mice all received bilateral i.c.v. injections of virus at P0. Mice injected with rAAV2/8-*UBQLN2* wild type and ALS-linked mutants all demonstrated widespread neuronal (specific) expression of ubiquilin-2 in the olfactory bulb, cortex, hippocampus, thalamus, striatum, brainstem, and cerebellum as early as 1 month post-injection, and maintained at both 3 and 6 month time points (Fig. 2). In sites near to the injection such as cortex, hippocampus, thalamus and striatum, nearly 30–40 % neurons were transduced. Western blots of whole brain tissue lysates similarly indicated sustained ubiquilin-2 expression through the 6 month time point with levels reaching 10–40 % that of endogenous mouse ubiquilin-2 (Fig. 3). Transduced neurons expressing human ubiquilin-2, however, were easily identified by immunohistochemistry relative to background endogenous mouse ubiquilin-2, suggesting several-fold overexpression. Expression of WT ubiquilin-2 in neurons was diffuse, involving the soma and proximal dendrites, and included few small punctate cytoplasmic accumulations (see Fig. 4, confocal images). In contrast, expression of each of the mutant forms of ubiquilin-2 resulted in large intracellular neuronal inclusions and extensive neuropil aggregates in the surrounding gray matter, similar to that recently described by Gorrie et al. in transgenic mice with the P497H mutant ubiquilin-2 [10]. Whereas WT ubiquilin-2 was mainly cytoplasmic and diffuse, mutant ubiquilin-2 expression also appeared to have more prominent nuclear localization. As early as 1 month dystrophic changes were also seen in the dendritic arbors of purkinje cells expressing mutant ubiquilin-2, which appeared to have reduced branching architecture (Fig. 2). Glial markers showed only a rare ubiquilin-2-positive astrocyte in areas of abundant viral expression (Fig. 4). Despite the presence of abundant large inclusion seen in mice expressing mutant forms of ubiquilin-2, there was no apparent neurodegeneration or cell loss even in 6 month mice. Tissues were immunostained for apoptotic cell markers including caspase-3/7 and tunnel stain, and both negative (data not shown). Examination of hematoxylin & eosin stained sections also showed no evident cell loss or degeneration of brain regions overexpressing ubiquilin-2.

### Mutant ubiquilin-2 inclusions colocalize with TDP-43 and are ThioS-positive

**Fig. 5:**
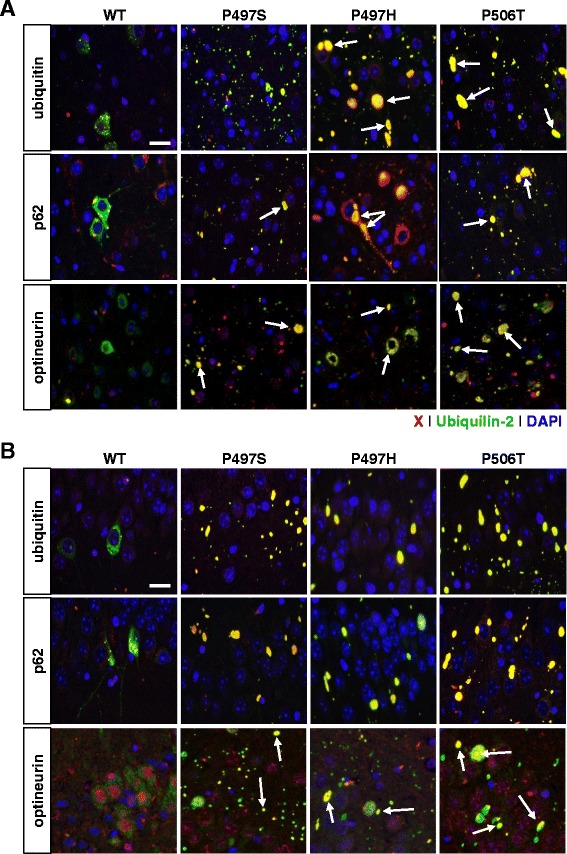
Ubiquilin-2 inclusions colocalize with ubiquitin, p62, and optineurin. Merged immunofluorescent images are from **a**) cortex and **b**) hippocampus from 6 month mice and show staining for ubiquitin/p62/optineurin (*red*), ubiquilin-2 (*green*), and DAPI (*blue*). In contrast to WT, pathological (mutant) ubiquilin-2 form large intracellular and neuropil inclusions that frequently colocalize (indicated as yellow, representing overlap *red* and *green* signal) with ubiquitin, p62, and optineurin. (bar = 25 μm)

**Fig. 6:**
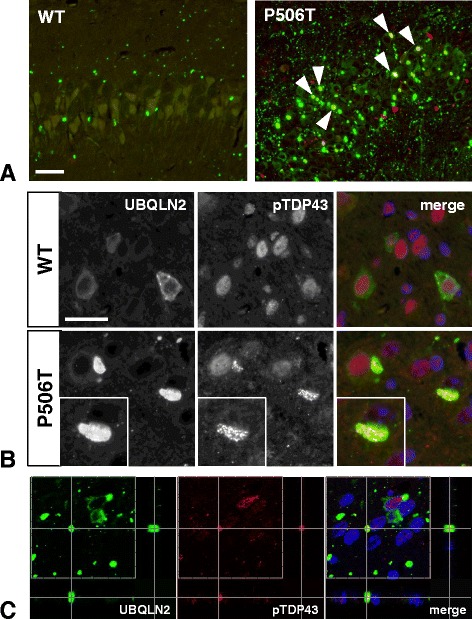
TDP43 colocalizes with ubiquilin-2 in mice expressing mutant UBQLN2 (P506T). **a**) Low power merged immunofluorescent images of CA3 hippocampus from mice injected with rAAV expressing WT or P506T mutant ubiquilin-2 and aged 6 months. TDP43 (*red*) colocalizes (*arrowheads*) with several ubiquilin-2-positive (*green*) inclusions in mutant P506T expressing mice. (bar = 25 μm) **b**) Higher power photomicrographs show cytoplasmic TDP43 puncta stained with the phospho-TDP43 antibody (403–404) within a large cytoplasmic ubiquilin-2-positive inclusion. (bar = 25 μm) **c**) Confocal Z-slice section analysis of ubiquilin-2 inclusions (*green*) similarly demonstrates colocalization of phosphorylated TDP43 (*red*) in brain tissue from mice expressing mutant UBQLN2 (P506T)

**Fig. 7:**
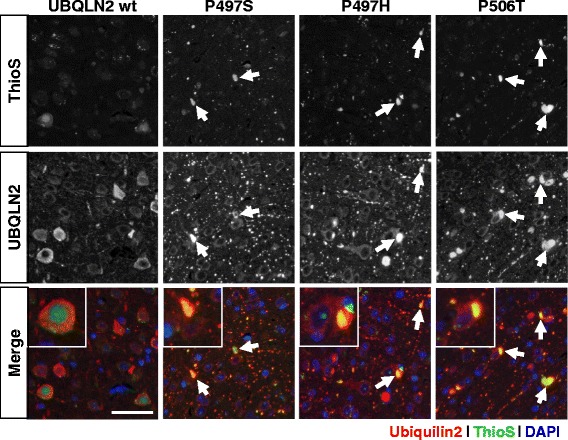
Mutant ubiquilin-2 expression induces ThioS-positive inclusions. Photos show Thioflavin-S staining that colocalizes (arrows; yellow in merged images) with intracellular ubiquilin-2-positive inclusions seen in mice injected with rAAV expressing mutant but not WT ubiquilin-2. Images shown are from mice aged 6 months, but similar findings were seen also in younger mice. (bar = 50 μm)

As ubiquilin-2 has been found colocalized with other proteins in ALS inclusions, such as ubiquitin, p62, and FUS [1, 14], we examined brain tissue from mice for co-localization of these and several other neuropathological proteins including pSer129-synuclein, tau, phospho-tau, and TDP-43, which is found both in frontotemporal dementia (FTLD-U) and ALS brains. Minimal differences were noted in expression patterns between 1, 3, and 6 month mice. As expected, ubiquilin-2-positive inclusions and aggregates co-stained for ubiquitin, p62, and optineurin (Fig. 5). However, ubiquilin-2 inclusions did not colocalize with FUS (except for that within nuclei) or phosphorylated α-synuclein using the pSer129/81A antibody, which has recently shown also to bind phosphorylated neurofilament subunit L, or NFL [15] (data not shown). Inclusions also did not colocalize with tau, consistent with published data that indicate no correlation of ubiquilin-2 with tau pathology [16]. However, in mice expressing the mutant ubuiqilin-2(P506T) cytoplasmic TDP-43 aggregates, immunostained with antibodies to either phospho-TDP-43 (403–404) or (409–410) epitopes, were associated with ubiquilin-2-positive inclusions (Fig. 6). These findings suggest that ubiquilin-2(P506T) may be more prone than the P497S/H mutants to cause proteinopathy involving TDP-43 pathology that is seen in frontotemporal dementia (FTD). Interestingly, expression of mutant forms and not WT, of ubiquilin-2 also resulted in inclusions or aggregates that stained positive for ThioflavinS suggesting induction of amyloid pathology (Fig. 7) further supporting the notion that ALS-linked ubiquilin-2 mutants induce proteinopathy via misfolding and aggregation of proteins.

### Viral SBT of ALS-linked *UBQLN2* mutants results in an early motor deficits

**Fig. 8:**
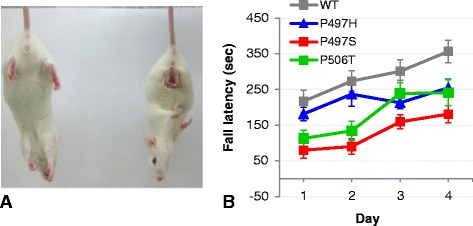
Behavioral deficits in mice. **a**) Mice expressing ALS-mutant ubiquilin-2 develop a clasping phenotype at 3–4 months. **b**) Rotarod performance for mice at 3 months expressing mutant ubiquilin-2 P497S (*p* < 0.0001) and P506T (*p* < 0.01) was significantly impaired compared to those expressing WT ubiquilin-2. Data shown as mean ± SEM; *N* = 9 for each group

So far in mice aged to 6 months we have not observed significant cell loss or neurodegeneration as determined by tunnel, caspase-3/7 or hematoxylin and eosin staining. However, at 3–4 months several mice expressing mutant ubiquilin-2 (P497S: 7 of 9, P497H: 2 of 9, and P506T: 4 of 9 mice) developed a nonspecific clasping phenotype (Fig. 8a). On rotarod testing mice expressing mutant ubiquilin-2 also showed significant impairment compared to mice expressing WT ubiquilin-2 (Fig. 8b). Despite the appearance of relative stable pathological features, these findings suggest progression of pathology and that more prolonged expression of ALS-linked ubiquilin-2 using our novel rAAV model system may result in a more disease-relevant motor phenotype.

## Discussion

*UBQLN2* mutations have recently been added to the list of potential genes that cause familial ALS and ALS-FTD [1, 2]. *UBQLN2* encodes for ubiquilin-2, a member of the ubiquitin-like family of proteins that facilitate transport of ubiquitinated proteins to the proteasome for degradation. Although evidence to date suggests that ALS-linked ubiquilin-2 mutants have reduced proteasomal function and cause a potential loss-of-function [1], the role of ubiquilin-2 in ALS pathology remains unclear. To determine the functional consequences of ALS-linked *UBQLN2* mutations, we developed rAAV vectors to express WT and three of the identified ubiquilin-2 mutants (P497, P497H, and P506T) in primary neuronal cells and in the developing mouse brain. In primary cultures we found that viral overexpression of ubiquilin-2 resulted in large intracellular accumulations that were more prominent and distributed along neuronal processes for mutant forms than for WT ubiquilin-2. Fractionated lysates from these cultures demonstrated also that mutant ubiquilin-2, but not WT, were present in TX-insoluble (SDS soluble) fractions, suggesting tendency for mutant forms of ubiquilin-2 to form insoluble aggregates. To determine whether viral expression ALS-linked mutant ubiquilin-2 could induce pathological and behavioral abnormalities in mice, we developed a model system using somatic brain transgenesis, or SBT, to widely and rapidly overexpress ubiquilin-2 in the developing mouse nervous system. We demonstrate herein that mice injected i.c.v. with rAAV-ubiquilin-2 mutants and aged up to 6 months develop early, widespread neuronal inclusion pathology, dystrophic neurite changes, and motor deficits.

To date few studies have examined the *in vivo* consequences of ALS-linked ubquilin-2 in brain and spinal cord. Recently, Gorrie et al. [10] published the first findings from transgenic mice that express one of the ALS-linked mutant ubquilin-2 (P497H) under the direction of the *UBQLN2* promoter. Progressive ubiquilin-2 pathology was observed in these mice and particularly prominent in the hippocampal gyrus, but also in the frontal and temporal lobes with increasing age, similar to that seen in human ALS tissues [1]. Abundant ubiqulin-2-positve neuropil aggregates in gray matter, but not in white matter, were noted [10] and similar to that observed in our mouse brains transduced with rAAV-UBQLN2 mutants. These findings suggest that ubiquilin-2 aggregates are localized to dendrites rather than axons. Indeed, electron microscopy studies indicate primary somatodendritic aggregates which are prominent in dendritic spines in hippocampal and cortical tissues and which may contribute to altered spine density and plasticity [10]. The findings from our mouse models are complimentary and together these models indicate that expression of ALS-linked ubiquilin-2 mutants cause progressive ubiquilin-2 pathology involving aggregate formation and proteinopathy. However, the link between these findings, neurodegeneration, and development of ALS remains unclear. In both our mouse SBT model and the *UBQLN2*^P497H^ transgenic mice, neuronal loss and neurodegeneration have not been observed. However, more recently, Wu et al. in a similar transgenic model in rats did show neuronal loss proceeded by formation of ubiquilin-2 aggregates and evidence of impaired autophagy and endosomal function [11]. Lack of evidence for neurodegeneration in our model may possibly be explained by relative low viral transduction of neurons (estimated at 30–40 %, greatest in regions near the ventricles); however, detailed analyses with both tunnel and caspase 3/7 were unrevealing. Nevertheless, in our study SBT mice expressing mutant UBQLN2 variably developed clasping and rotarod deficits as early as 3–4 months, which although nonspecific may indicate progressive pathology and possible later development of a more disease-relevant motor phenotype. This finding is in contrast to recent transgenic P497H models that report evidence for cognitive rather than motor deficits [10, 11], which may have relevance to ALS-FTD and other neurodegenerative dementias. To fully determine the utility of our novel rAAV model system, we will need to further establish the effects of mutant ubiquilin-2 expression in mouse brain and spinal cord beyond 6 months to determine whether we can induce pathological and phenotypic changes, such as paresis, expected for ALS/ALS-FTD.

Evidence to date indicates that ubiquilins play important roles in multiple protein recycling and degradation pathways, including the UPS, ERAD, and autophagy [17]. Although the function of ubiquilin-2 remains unclear, its homology to ubiquilin-1 suggests a similar function and role in the UPS and degradation of proteins. Identified ALS-linked mutations in ubiquilin-2 all localize to a proline-rich (PXX repeat) region that is distinct from either the N-terminal UBL (ubiquitin-like) domain that interacts with the proteasome or the C-terminal UBA (ubiquitin-associated) domain that associates with ubiquitinated proteins, suggesting that ALS mutants may leave these functional domains intact. ALS-linked mutations in ubiquilin-2 have been shown *in vitro* to impair proteasomal degradation and these findings appear consistent with its primary function in the UPS [1]. Recently *in vivo* data from bigenic mice expressing both *UBQLN2*^P497H^ and the ubiquitinated protein substrate, *Ub*^G67V^-GFP, appears to support these findings. *Ub*^G67V^-GFP accumulated in the brain of bigenic mice expressing *UBQLN2*^P497H^ suggesting impaired UPS function [10]. Furthermore, ubiquilin-2 deposits in brain sections from these mice colocalized with antibodies to proteasome subunits. These findings appear to indicate that mutant ubiquilin-2(P497H) may still function to bring ubiquitinated proteins to the proteasome, but somehow interferes with proteasomal degradation, leading to accumulation and abnormal deposition proteins. Our data indicate that ALS-linked ubiquilin-2 variants may have differential effects on UPS function. Indeed the P497H mutant had little effect on d2EGFP levels and was similar to WT, whereas expression of both the P497S and P506T mutants impaired d2EGFP metabolism (Fig. 7). These data suggest that alternative protein degradation mechanisms may be involved such as the autophagy-lysosomal system to explain the effects of these ubiquilin-2 mutants on proteinopathy seen in our models.

Recent studies also implicate ubiquilin-2 in macroautophagy. Early studies demonstrated that ubiquilin-1 binds the target of rapamycin (mTOR) kinase in mammalian cells, a critical regulator of macroautophagy [18]. Both ubiquilin-1 and 2 have also been shown to colocalize with the microtubule-associated protein 1 light chain 3 (LC3), a membrane component of autophagosomes, and have been implicated in the maturation of autophagic vesicles [5]. Notably, knockdown of ubiquilin-2 (and 1) rendered cells expressing either a Alzheimer’s-related presenilin mutant or a huntingtin polyglutamine expansion more susceptible to starvation-induced death, whereas overexpression is protective, further supporting a role in autophagy and neurodegenerative disease [5]. The effects of ALS-linked mutations on ubiquilin-2 function in macroautophagy have not been explored and remain unclear. We hypothesize that expression of ubiquilin-2 mutants may impair macroautophagy, as well as UPS function, disrupting proteostasis and contributing to protein accumulation, aggregate formation, cell stress and cytotoxicity.

To date, few studies have identified protein interactors with ubiquilin-2 or ALS-linked mutants. UBA and UBL domains in ubiquilins are known to interact with polyubiquitinated proteins and the proteasome, respectively, consistent with their function in the UPS [4]. In addition, ubiquilins have been shown to interact with components of the ERAD including Erasin and p97/VCP (valosin-containing protein) that form a complex at the ER membrane to direct degradation of misfolded protein as part of the unfolded protein response [6]. More recently, ubiquilin-2 was shown to interact with the ubiquitin regulatory X domain-containing protein 8 (UBXD8), which mediates translocation of ERAD substrates such as p97/VCP, and this interaction was impaired by the ubiquilin-2 mutant (P497) [19]. Although ubiquilin-2 has been colocalized with several other proteins *in vitro* and *in vivo* including LC3 [5], p62/SQSTM1, ubiquitin [1], and optineurin [10], direct interactions have not been demonstrated. Our data indicate colocalization of ubiquilin-2 inclusions with cytoplasmic, phospho-TDP-43 in mice expressing the P506T mutant ubiquilin-2. Recent evidence suggests that ubiquilin-2 binds to C-terminal fragments of TDP-43 [1, 20]. TDP-43 and in particular mislocalization and aggregation of C-terminal fragments of TDP-43 have been implicated in both ALS and FTD pathology. Together, these data provide an incomplete picture of proteins that may interact with ubiquilin-2 or ALS-linked mutants that may be critical to understanding both the normal function of ubiquilin-2 as well as how identified mutations alter its function and may influence development of ALS/ALS-FTD pathology.

## Conclusions

We demonstrate using rAAV techniques that overexpression of ALS-linked mutant *UBQLN2* induce pathological accumulations of ubiquilin-2 in neurons, insoluble aggregates, and early behavioral deficits in our SBT mouse model. Our findings lend support to the notion that mutant ubiquilin-2 expression result in a (toxic) gain of function, disrupting proteostasis. Although traditional transgenic approaches are being used to investigate the pathological consequences of ubiquilin-2 mutant expression in mice [21], we report here the first use of a novel somatic brain transgenic approach using rAAV serotype 8 that shows a similar pattern of widespread neuronal inclusion pathology in brain. This approach has several advantages in that we are able to relatively rapidly test several of the recently reported ubiquilin-2 variants and highlight potential differences in their pathological effects, as well as noted a potential relevant motor phenotype not previously reported. Clearly there are several limitations to SBT including variability among injections and limited viral expression. However, the use of rAAV provides agility to easily modify future constructs to test specific portions of the ubiquilin-2 that may differentially affect aggregation and pathology or to express in select cell types to address possible non-cell autonomous effects suggested in ALS [22].

## Methods

### Cloning and rAAV preparation

Both WT and mutant *UBQLN2* (P497S, P497H and P50T) constructs were generated using PCR and were subcloned into recombinant adeno-associated viral (rAAV) vectors, serotype 2, with expression cassette containing a cytomegalovirus enhancer/chicken beta actin (CBA) promoter, bovine growth hormone polyA, and woodchuck hepatitis virus post-transcriptional regulatory element (WPRE). AAV control vector expressing EGFP was prepared as previously described by Chakrabarty et al. [13]. Recombinant AAV constructs were packaged into AAV with serotype 2/8 capsid using methods derived from Zolotukhin et al. [23]. Briefly, we co-transfected rAAV into HEK293T cells with linear polyethylenimines (PEI, Polysciences) along with AAV helper plasmid 8 (Plasmid Factory, Germany). Cells were harvested, lysed, and virus isolated with an iodixanol gradient, and then buffer exchanged to sterile PBS, pH 7.2. Viral titers (genome copies per mL) were determined by quantitative PCR (Bio-Rad, CFX384) as previously described and [13]. AAV titers were as follows: *UBQLN2* WT 2.30×10^13^ gc/mL, P497S 1.38x10^13^ gc/mL, P497H 1.30×10^13^ gc/mL, P506T 1.26×10^13^ gc/mL, and EGFP 2×10^13^ gc/mL. All freshly prepared AAVs were aliquoted and stored at −80 °C until use. *Neuroglial cultures*. Primary mixed neuronal-glial cultures were prepared as previously described by Sacino et al. [24]. Briefly, mouse cortices from B6C3HF1 mice were isolated at E16. The tissue was dissociated by digestion with papain solution (Worthington Biochemical Corp, NJ) and 50ug/ml DNase I (Sigma, MO) at 37 for 20 min. After digestion cortices were washed three times with Hank’s Balanced Salt Solution (HBSS, Life Technologies) to remove the papain and place in media consisting of Neurobasal (Life Technologies) supplemented with 0.02 % NeuroCult™ SM1 (STEMCELL Technologies Inc., Vancouver), 0.5 mM GlutaMax (GIBCO, Life Technologies), 5 % Fetal Bovine Serum (Hyclone, GE Life Sciences) and 0.01 % Pen-strep (GIBCO, Life Technologies). The tissue was triturated in the same media and dissociated cells were plated in CC2-coated cell Lab-Tek II 8-chamber slides (Fisher Scientific) at a density of 20,000 cells per well for imaging and in poly-D-lysine (Sigma, MO) coated 6-well plates for biochemical analysis. Cells were maintained at 37 °C in a humidified incubator with 5 % CO_2_.

#### Double Immunofluorescence analysis of mixed neuroglia cultures

Cells were transduced at DIV-6 (days *in vitro*) with rAAV2/8 *UBQLN2* WT and mutants to a final concentration of 10^11^ gc/ml. At DIV-10 cells were fixed with 4 % paraformaldehyde in PBS (0.01 M phosphate buffered saline, pH 7.4), then washed with PBS and blocked in 5 % goat serum with 0.1 % triton X-100 in PBS for 1 h, and then incubated overnight in primary antibodies: UBQLN2 (1:500; Abcam) and MAP2 (1: 1000; Abcam). Cells were washed in PBS and then incubated in secondary antibody goat-anti mouse conjugated to Alexa-488 and goat anti-rabbit conjugated to Alexa-594 (1:1000; Life technologies). Nuclei were counterstained with mounting media containing 4′,6-diamidino-2-phenylindole (DAPI). Images were captured using Olympus BX-60 epi-fluorescence microscope with DP71 digital camera.

#### Biochemical fractionation followed by western blot analysis

Cells for Western blot were extracted using TBS (tris-buffered saline) and 1 % triton X-100 supplemented with proteinase and phosphate inhibitors (TBS-T buffer), vortexed, and incubated on ice for 5 min. Tissue samples from adult mouse brain were weighed (wet weight), then digested mechanically in 4× volume of same lysis buffer (ice-cool), and similarly incubated on ice for 5 min. Lysates were centrifuge at 100,000 g for 20 min at 4 °C, the supernatant saved (soluble fraction), and the pellets re-washed with TBS-T buffer and re-centrifuged with the same buffers to remove any trace of the soluble fraction. The insoluble fractions were the extracted from the remaining pellets using 2 % SDS (sodium dodecyl sulfate) and sonication. Equal amounts of Soluble and Insoluble fractions were visualized by SDS protein electrophoresis and detected by mouse monoclonal UBQLN2 antibody (1:1000; Abcam). Ubiquilin-2 was normalized to actin (AC15, Sigma) in blots.

#### Proteasomal assay

HEK293 cells were transfected with a UPS reporter vector encoding d2EGFP. 24 h post transfection, cells were re-plated into 12-well plates and transfected with either wild type or mutant ubiquilin-2. 24 h after second transfection, cells were treated with 30 μg/ml of cyclohexamide (Sigma) for 0, 3, 6, 9, or 12 h. At each time point, cells were harvested, washed in ice-cold PBS and lysed in RIPA buffer including protease inhibitors. Equal amounts of protein were loaded for Odyssey blotting, and the d2EGFP levels were normalized to β-actin. Data were collected from three independent experiments.

#### Mice, neonatal injections, and behavioral assessment

All animal husbandry and procedures were approved by the University of Florida Institutional Animal Care and Use Committee and conformed to the NIH guidelines for animal research. B5C3HF1 and FVB mice were obtained from Harlan labs (Tampa, FL) for use in these studies. Neonatal mice were kept with parent mother until weaned. Mice were otherwise housed three to five per cage, given food and water *ad libitum*, and kept on a 12 h light/dark cycle.

AAV were injected in newborn mice P0 (0–24 h old) as described in Chakrabarty et al. [13]. Briefly, rAAV-UBQLN2 were delivered to non-transgenic FVB mice via bilateral intracerebroventricular (i.c.v.) injections. Each injection included 2 μL rAAV (1–3x10^13^ gc/mL) expressing UBQLN2 WT or P497S, P497H, P506T mutant or EFGP (control) into both cerebral ventricles. For each virus, approximately 12–18 mice were injected (2–3 litters). Mice were observed and underwent periodic SHIRPA primary screen testing [25]. At 3 months a subset of mice (*n* = 9 per group) performed rotarod testing. Mice were sacrificed at set timepoints: 1 month (*n* = 2–3 per group), 3 months (*n* = 6–8) and 6 months (*n* = 6–8) post-injection. Animals were euthanized by CO_2_ inhalation, briefly perfused transcardially with PBS, and brains harvested immediately. Half of the brain was fixed in 10 % formalin, washed and embedded in paraffin for sections; the other half was flash-frozen for later biochemical analysis.

#### Rotarod testing

Mice were trained in groups of 3–5 on a Rotamex-5 apparatus (Columbus Inst., OH). Mice were given a series of pre-training trials the day before testing, including 3, 5 min runs on the rotarod at constant speed (5 rpm). During the following 4 consecutive days, mice were tested with 4, 5 min trials (40–60 min inter-trial interval) with gradual acceleration of the rod from 4 to 40 rpm. The speed and latency to fall were recorded for each trial. Best performances from each of the 4 test trials on each consecutive day were analyzed and groups compared using repeated measures ANOVA.

#### Immunohistochemistry and immunofluorescence analysis of brain sections

Paraffin embedded brains were cut into 8 μm sagittal sections. Sections were deparaffinized and dehydrated in xylenes and serial alcohol concentrations (70–100 %) followed by water antigen retrieval, steam, or retrieval solution (Dako) for 30 min followed by hydrogen-peroxide incubation. Sections were immunostained with primary antibody to UBQLN2 (Sigma; 1:500) and other specific antibodies (as listed below) overnight, and then developed using Immpress polymer detection reagents (Vector Labs). Sections were counterstained using hematoxylin solution. Separate sections also underwent hematoxylin and eosin staining. Brain images were scanned using ScanscopeXT image scanner (Aperio/Leica USA). For double immunofluorescence sections were immunostained with primary antibody to UBQLN2 (5 F5, Novus Biologicals; or HPA006431, Sigma-Aldrich) in combination with other antibodies including: ubiquitin (Ab7780, Abcam, Cambridge, MA), p62 (SQSTM1, Proteintech, Chicago, IL), GFAP (Dako, Carpinteria, CA), Iba-1 (Abcam), MAP2 (Abcam), caspase 3, α-synuclein (Syn1, BD Biosciences, San Jose, CA), pSer129-synclein [26], NFL (neurofilament, C28E10, Cell Signaling Technologies; or monoclonal NR4, Sigma-Aldrich), PHF1 (provided by Dr. Peter Davis), TDP-43 (Cosmo Bio, Carlsbad, CA), phospho-TDP43 (403/404 and 409/410 antibodies, Cosmo Bio), FUS (Bethyl, Montgomery, TX), Matrin-3 (2539C3a, Abcam), Optineurin (Abcam), and VCP/p97 (Abcam). For visualization fluorescent conjugated antibodies, Alexa 594-goat anti-mouse or anti-rabbit and Alexa 488-goat anti mouse at 1:500, were used. Fluorescent images were captured using either Olympus BX60 microscope with epi-fluorescence, confocal spinning disc (Olympus DSU-IX81) or laser confocal microscope (Leica TCS SP2 AOBS spectral) for analysis.

## Abbreviations

ALS: Amyotrophic lateral sclerosis
ERAD: Endoplasmic reticulum associated degradation
FTD: Frontotemporal lobar dementia
FUS: Fused in sarcoma
icv: Intracerbroventricular
NFL: Neurofilament light chain
rAAV: Recombinant adeno-associated virus
SBT: Somatic brain transgenesis
TDP-43: Transactive response DNA binding protein 43
UBA: Ubiquitin binding domain
UBQLN2: Ubiquilin-2
UBL: Ubiquitin-like domain
UPS: Ubiquitin-proteasome system
